# Using Piezosurgery in Anterior Cervical Discectomy and Fusion to Treat Complex Cervical Spondylotic Myelopathy Is Safe and Effective

**DOI:** 10.1155/2023/5306445

**Published:** 2023-12-21

**Authors:** Yu-Wei Li, Hao-Jie Chen, Shi-Xin Zhao, Xiu-Zhi Li, Hai-Jiao Wang, Peng Zhou, Wei Cui, Wei Xiao, Fan Li, Bingtao Hu

**Affiliations:** ^1^Department of Orthopedics, Luohe Central Hospital, Luohe, Henan 462000, China; ^2^Medical College, Zhengzhou University, Zhengzhou, Henan 450052, China

## Abstract

**Objective:**

To investigate the safety and efficacy of piezosurgery in anterior cervical discectomy and fusion (ACDF) for cervical spondylotic myelopathy (CSM).

**Methods:**

47 patients with complex CSM (cCSM) underwent ACDF surgery from 2014 to 2017. Among these patients, 26 underwent ACDF using piezosurgery (group A) and 21 underwent ACDF by using traditional tools such as high-speed air drill, bone curette, and Kerrison bone punch (group B). Average surgical time, intraoperative blood loss, surgical complications, preoperative and postoperative Japanese Orthopaedic Association (JOA) scores, and improvement rate were measured.

**Results:**

Average surgical time and intraoperative blood loss were significantly lower in group A than those in group B (*P* < 0.01). The incidences of surgical complications were 3.8% and 23.8% in the A and B groups (*P* < 0.05), respectively. There were no significant differences in JOA scores and improvement rates between data collection periods at preoperative, 3-day postoperative, and 1-year postoperative follow-ups (*P* > 0.05).

**Conclusion:**

For treating cCSM, both the piezosurgery and traditional tools led to significant neurological improvement. However, the piezosurgery was superior to the traditional tools in terms of surgical time, blood loss, and complication rate. Hence, piezosurgery was a safe and effective adjunct for ACDF treating cCSM.

## 1. Introduction

Cervical spondylotic myelopathy (CSM) is characterized by progressive degeneration of the vertebral body and ligaments, resulting in spinal stenosis and subsequent compression of the spinal cord [1, 2]. Surgery should be considered if conservative treatment fails. Anterior cervical discectomy and fusion (ACDF) is an effective method for the treatment of CSM [3, 4]. However, for patients with (1) large retrovertebral body osteophytes adjacent to the endplate or (2) a free nucleus pulposus that migrated to the vertebral body, posteriorly, known as complex CSM (cCSM) here, a surgeon may consider anterior cervical corpectomy and fusion (ACCF) [5, 6]. In this case, ACDF may be suboptimal due to the higher chance of incomplete decompression posterior to the midvertebral body and limited visual access during the surgery [5, 7]. A majority of studies have endorsed the clinical outcomes of ACCF treatment for cCSM. Some studies have indicated, however, that ACCF is associated with a higher incidence of complications including a wider range of operations and an increased risk of plate implant extrusion due to the fewer possible points for ventral plate screw fixation [8, 9].

Traditional surgical instruments such as high-speed drills and Kerrison bone punch can have a negative impact on bone healing. Given that piezosurgery has been used in spinal, oral, and maxillofacial surgery, surgeons using piezosurgery in vertebroplasty have found that it can reduce intraoperative bleeding and dural injuries when compared to high-speed drills, while achieving similar clinical outcomes [10, 11]. The authors found that if cCSM is caused by a soft free nucleus pulposus that does not exceed 1/2 of the height behind the adjacent vertebral body or a bone hyperplasia that does not exceed 1/3 of the height behind the attached vertebral body, can ACDF be used for complete decompression through the narrow vertebral space using piezosurgery? If that hypothesis is confirmed, the application of ACCF is reduced to minimize the risk of plate implant extrusion, shorten the fusion distance, and decrease the risk of nerve damage.

To date, the safety and efficacy of ACDF in the treatment of cCSM with piezosurgery are unknown. Moreover, few studies have compared clinical outcomes between piezosurgery and traditional tools in ACDF for cCSM. The purpose of this study was to assess the differences in perioperative and 1-year postoperative outcomes associated with piezosurgery versus traditional tools for the treatment of cCSM.

## 2. Materials and Methods

### 2.1. Patients' Information

**Figure 1:**
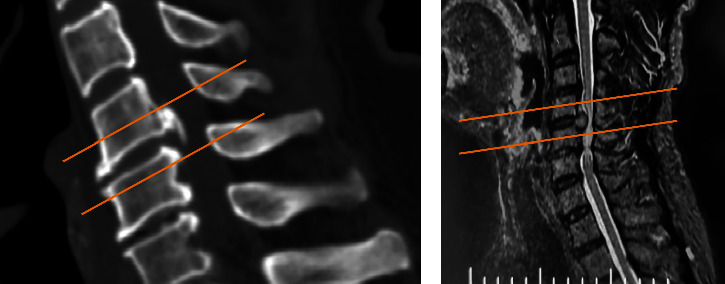
Case inclusion criteria: (a) osteophytes not more than 1/3 of the vertebral body height or (b) nucleus pulposus should not exceed half of the vertebral body height.

Forty-seven consecutive patients were treated with ACDF for single-segment cCSM in our institution from 2014 to 2017. Among these patients, 26 were treated using piezosurgery for decompression (group A), whereas 21 were subjected to traditional tools such as high-speed air drill, bone curette, and Kerrison bone punch (group B). Inclusion criteria: (1) diagnosed as CSM, including medical history, physical examination, and imaging examinations. (2) Free nucleus pulposus and/or posterior osteophytes of the vertebral body are limited to one segment. (3) The lesions were between C_3/4_ and C_6/7_. (4) Free nucleus pulposus not exceeding 1/2 of the posterior height of the vertebral body and osteophytes not exceeding 1/3 of the posterior height of the vertebral body to which they are attached. Exclusion criteria: (1) patients with 2 or multilevel cCSM (≥2 levels). (2) Those who had a previous history of cervical surgery were excluded (Figure 1). Patients were divided into two groups according to intraoperative decompression tools: group A (26 cases) using piezosurgery and group B (21 cases) using traditional tools.

Approval from the Institutional Review Board was obtained. Patients' outcomes were initially collected independently from patients with informed consent and then analyzed blindly to avoid influencing the outcome scores.

### 2.2. Surgical Technique

All patients took the supine position under general anesthesia. The right transverse incision was taken and separated until the vertebral body was exposed. After removing the intervertebral disc, the intervertebral space was opened.

#### 2.2.1. Group A

**Figure 2:**
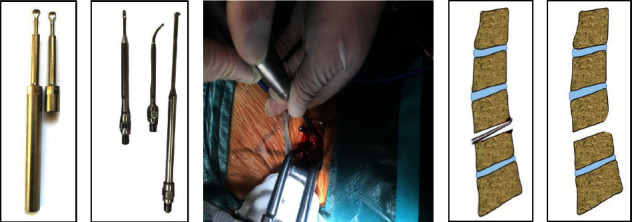
Diagram of piezosurgery operation: (a) straight piezosurgery, (b) piezosurgery with different angles and shapes, (c) piezosurgery was used to remove osteophytes from the posterior margin of the vertebral body, and (d, e) piezosurgery was used to remove osteophytes behind the vertebral body until the posterior intervertebral space became a trapezoid with a narrow front and wide back.

All patients were treated with piezosurgery for decompression. First, the cortical bone and/or osteophytes at the posterior edge of the vertebral body were scraped with a long-handled straight curet, and then the curved curetwas attached to the posterior longitudinal ligament (PLL) and placed in the posterior edge of the vertebral body, which was further scraped until the posterior margin of the intervertebral space formed a trapezoid which was narrow in front and wide in back. The PLL attachment on the vertebral body was disconnected in this enlarged space, and this was followed by the detection and removal of the compressors in front of the spinal dural. The schematic of ACDF using piezosurgery is shown in Figure 2.

#### 2.2.2. Group B

All patients had the posterior edge of the vertebral body thinned with the high-speed air drill, the bone cortex and/or osteophytes at the posterior margin of the vertebral body were then removed using a conventional bone curette or Kerrison bone punch, and the inverted trapezoid of the intervertebral space was considered as complete decompression. The PLL was removed, and the compressors were explored and removed under direct vision.

In the 2 groups, zero-P was implanted in the intervertebral space after complete decompression and the incision was closed after intraoperative anteroposterior and lateral X-ray confirmed that the plate implants were in good position. All the patients were allowed to get out of bed the next day after the operation and perform limb rehabilitation exercises.

### 2.3. Outcome Measures

Observation indicators included general data, neurological function indicators, and radiological data.

The general indicators consist of the operation time, intraoperative blood loss, and surgical complications including spinal cord or nerve root injury, cerebrospinal fluid leakage, injury of the superior laryngeal nerve or recurrent laryngeal nerve, dyspnea, dysphagia, hematoma, C_5_ nerve root paralysis, infection of the surgical incision, and pulmonary and urinary tract infections.

The nerve functions were evaluated by the JOA score, and the neurological function improvement rate was calculated as follows:

(1) $$\begin{array}{c} t\text{he improvement rate}=\frac{\text{follow}‐\text{up score}-\text{preoperative score}}{17‐\text{preoperative score}}\times 100\%. \end{array}$$

The radiological indicators including X-ray, computed tomography (CT), and magnetic resonance imaging (MRI) were reviewed for assessing the preoperative, postoperative, and final follow-up status and for evaluating the degree of preoperative compression and the effect of postoperative decompression as well as the plate implant position.

### 2.4. Statistical Analysis

SPSS 19.0 software was used for statistical analysis. Measurement data were expressed as the mean ± standard deviation, and a *t*-test was used to compare the operation time, intraoperative bleeding, and JOA score and improvement rate between the two groups, and the difference was considered statistically significant at *P* < 0.05.

## 3. Results

### 3.1. General Indicators

**Figure 3:**
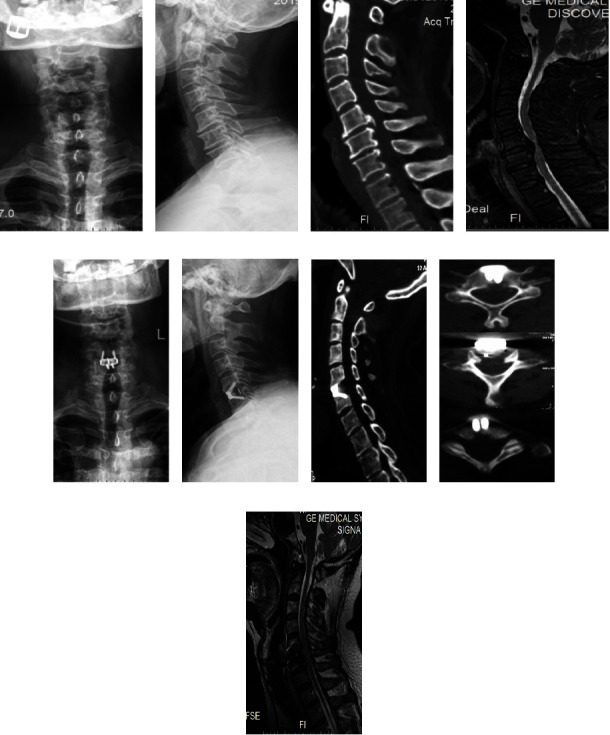
56-year-old man with CSM treated with ACDF using piezosurgery: (a, b) preoperative anteroposterior and lateral X-ray images showed the presence of large osteophytes in the C_5/6_ vertebral bodies, (c) preoperative sagittal CT examination showed C_5/6_ intervertebral disc herniation with calcification, (d) preoperative sagittal MRI revealed the C_5/6_ disc herniation that compressed the spinal cord, (e, f) postoperative anteroposterior and lateral X-ray examination showed that the plate implants were in good position and the posterior vertebral osteophytes had disappeared, (g, h) postoperative sagittal CT showed that C_5/6_ posterior osteophytes were removed completely, and the cervical canal area was significantly increased, and (i) postoperative sagittal T2-weighted MRI showed relief of C_5/6_ spinal cord compression.

There were no significant differences in sex, age, BMI, involved lesions, and Japanese Orthopaedic Association (JOA) score between the two groups before surgery (*P* > 0.05). The surgeries of all patients in the 2 groups were completed successfully. The last follow-up was set at 1 year postoperatively. In terms of the demographic data of the two groups, no significant statistical difference was found (Table 1). Typical case data are shown in Figure 3.

The operation time was 45.7 ± 3.9 min and 52.7 ± 6.7 min in group A and group B, respectively. In group A, the intraoperative blood loss was 48.9 ± 4.4 ml, compared with the intraoperative blood loss of 117.9 ± 16.3 ml in group B. There were significant differences between the two groups in the operation time and intraoperative blood loss (*P* < 0.05). In terms of complications, all implants were correctly positioned and not loosened or dislodged, only 1 patient in group A had a urinary tract infection, and no cerebrospinal fluid leakage or spinal cord injury occurred, while 2 patients in group B had cerebrospinal fluid leakage, 1 had hoarseness, and 2 had swallowing discomfort and no spinal cord injury occurred. A statistically significant difference was found between the two groups (*P* < 0.05) (Table 2).

### 3.2. Neurological Function Indicators

The JOA score in group A was 7.2 ± 0.9 preoperatively, 11.3 ± 1.2 postoperatively (JOA improvement rate of 39.0%), and 14.6 ± 1.8 at final follow-up (JOA improvement rate of 71.6%); in group B, the JOA score was 7.4 ± 1.0 preoperatively, 11.6 ± 1.1 postoperatively (JOA improvement rate of 38.7%), and 14.6 ± 1.9 (JOA improvement rate of 69.4%) at final follow-up. There was no significant difference between the two groups in the JOA scores and JOA improvement rate for neurofunctional improvement at all time points (*P* > 0.05). However, the postoperative and the last follow-up were significantly improved in both groups compared with the preoperation (*P* < 0.001) (Table 2).

## 4. Discussion

Anterior cervical surgery, including ACDF and ACCF, is the mainstream surgical method for the treatment of CSM with localized compression lesions [12–14]. Both of them can effectively remove intervertebral discs and retrovertebral body osteophytes [9, 15]. The advantages of ACDF are the ability to reduce damage to normal structures of the cervical vertebra while fully decompressing and less intraoperative bleeding. Nevertheless, due to the limitation of ACDF in the narrow operating space, confined surgical field, and difficulty in hemostasis, the risk of spinal cord injury is higher than that of ACCF, especially in the patients with CSM coincident with large posterior osteophytes adjacent to the endplate or a free nucleus pulposus that migrated to the rear of the vertebral body [16]. Therefore, in consideration of safety and decompression, most researchers adopted ACCF for patients with large osteophytes or free nucleus pulposus in the posterior vertebral body. One main advantage of piezosurgery is that it can easily remove the bone of the vertebral body until the intervertebral space presents a trapezoidal shape with a narrow front and wide back, which expands the surgical field and operating space, and then can dislodge osteophytes and free nucleus to relieve spinal cord compression; therefore, ACDF can be performed on the abovementioned patients safely and effectively [17]. As a revolutionary surgical tool in the field of spinal surgery, some clinical studies have reported that ultrasonic osteodynamic systems can improve surgical efficiency, shorten the surgical time, reduce bleeding, and ensure neurosafety [12, 18, 19]. Even when large osteophytes and free nucleus pulposus are encountered, cCSM could still be treated with ACDF instead of ACCF with the aid of piezosurgery.

Traditional decompression tools including conventional bone curette and Kerrison bone punch forceps have the potential risk of squeezing the dura, while high-speed air drills have the risk of scraping the surrounding soft tissue and damaging the spinal dural [20–23], as these may cause spinal cord injury, one of the most serious complications of anterior cervical surgery. Piezosurgery's mechanical vibration (amplitude 0.05∼0.36 mm and frequency of 22.5 kHz ∼ 40 kHz) generates a cutting effect that can effectively cut the bone, while its amplitude is less than the elastic limit of the dura mater, the dura mater can absorb the energy generated by piezosurgery's mechanical vibration through elastic vibration, thereby avoiding damage to the spinal cord [6, 24, 25]. Some studies also supported the abovementioned views in which Nakagawa showed that the use of piezosurgery could reduce the occurrence of mechanical injuries of the spinal cord [26], while Nakase confirmed that the ultrasonic dynamic system was more suitable for the operation of delicate structures [27]. In this study, osteophytes at the posterior edge of the vertebral body were removed by using a piezosurgery to make the intervertebral space into an inverted trapezoidal shape, fully relieving the spinal cord compression and ensuring safety at the same time. No device-related dural tear or spinal cord injury was found in patients in group A; however, 2 patients had cerebrospinal fluid leakage in group B.

In addition to the neuroprotective advantages, the reduction of intraoperative bleeding is another advantage of piezosurgery. Sanbom found that the ultrasonic dynamic system significantly reduced the amount of blood loss in the process of bone cutting compared with traditional tools, based on its ultrasonic cavitation effect [18, 19]. The results of this study showed that the surgical time and intraoperative blood loss in group A were significantly less than those in group B.

Several limitations of this retrospective study should be noted. The small sample of patients in our study, being monocentric, may have affected the results of this study. Moreover, the last follow-up time of all patients was relatively short. Polycentric data with a larger sample of patients are urged to further clarify the safety and efficiency of piezosurgery.

## 5. Conclusion

Studies have demonstrated that the use of piezosurgery can be used to perform ACDF in cCSM patients with large osteophytes or free nucleus pulposus, whereas with conventional surgical instruments, these patients usually require ACCF surgery with more implant-related complications. Although either the piezosurgery or traditional tools resulted in significant neurological improvement, nevertheless the former was superior to the latter in terms of operative time, intraoperative blood loss, and surgical complications. The piezosurgery may be a better candidate for treating cCSM combined with large osteophytes or free nucleus pulposus compared with the traditional tools.

## Figures and Tables

**Table 1:** Comparison of general data between the two groups ($\bar{x}\pm s$).

	Number	Gender		Age (years)	BMI	Surgical level			
		Male	Female			C_3/4_	C_4/5_	C_5/6_	C_6/7_
Group A	26	15	11	54.00 ± 9.75	24.12 ± 5.06	2	7	11	6
Group B	21	11	10	49.33 ± 8.05	24.26 ± 5.45	2	6	8	5
Statistics value		*χ* ^2^ = 0.133		*t* = 1.761	*t* = 0.091	*χ* ^2^ = 0.111			
*P* value		0.716		0.085	0.928	0.991			

BMI, body mass index.

**Table 2:** Comparison of two groups' operation times, intraoperative blood loss, neurological functions, and complications ($\bar{x}\pm s$).

	Number	Surgical time (min)	Intraoperative blood loss (ml)	Complications	JOA scores				JOA improvement rate	
					Preoperative	Postoperative	Last follow-up	*F* and *P* values	Postoperative	Last follow-up
Group A	26	45.70 ± 3.93	48.85 ± 4.38	1	7.20 ± 0.91	11.31 ± 1.20	14.61 ± 1.80	*F* = 195.549 *P* < 0.001	39.0%	71.6%
Group B	21	52.74 ± 6.71	117.86 ± 16.32	5	7.38 ± 1.00	11.55 ± 1.08	14.57 ± 1.89	*F* = 143.315 *P* < 0.001	38.7%	69.4%
Statistics value		*t* = 4.254	*t* = 18.835	*χ* ^2^ = 4.157	*t* = 0.611	*t* = 0.703	*t* = 0.061		*χ* ^2^ = 0.686	*χ* ^2^ = 1.231
*P* value		<0.001	<0.001	0.041	0.544	0.485	0.951		0.496	0.225

JOA, Japanese Orthopaedic Association.

## Data Availability

The data used to support the findings of the study are included within the article.
